# Recurrent endometrial stromal sarcoma in the abdominal wall following a total hysterectomy: A rare case report

**DOI:** 10.1016/j.ijscr.2020.03.003

**Published:** 2020-03-07

**Authors:** Sigit Purbadi, Kade Yudi Saspriyana

**Affiliations:** Gynecology Oncology Division, Department of Obstetrics and Gynecology, Faculty of Medicine, Universitas Indonesia, Dr. Cipto Mangunkusumo National Referral Hospital, Indonesia

**Keywords:** Case report, Endometrial stromal sarcoma, Reconstruction, Recurrent, Wide excision

## Abstract

**Introduction:**

High-grade endometrial stromal sarcoma (HG-ESS) is a rare pathological type of uterine sarcoma. Over 80 % of affected patients would experience recurrences within a few years of initial presentation. Such case is rare and therefore, we need to report the case including the management. Information on performing good surgical techniques is important.

**Case presentation:**

A 55-year-old female patient was referred after having a total hysterectomy and bilateral salphingoophorectomy at a private hospital. Results of pathologic examination showed that the patient had HG-ESS. The patient subsequently received adjuvant chemotherapy. The regimens used were Carboplatin (AUC-6) and Paclitaxel (175 mg/m^2^). Within five months following the chemotherapy, she complained rapid abdominal enlargement, which was a clinically mobile palpable solid mass at the level of the navel. Imaging findings suggested recurrent endometrial stromal sarcoma in the abdominal wall. Wide excision and frozen section, which were continued with mesh insertion and abdominal wall reconstruction, were then performed.

**Discussion:**

Recurrence develops in one-half to two-third of patients with HG-ESS. It has been reported that the sites of recurrence usually include multiple lung metastases, peritoneal metastases, and/or local recurrences. There is currently no standard therapy for patients with recurrence of the disease as HG-ESS is a rare clinical entity. Treatment has been defined based on experiences gained from retrospective case reports.

**Conclusion:**

Successful management of patients with recurrent abdominal wall HG-ESS requires interdisciplinary and interprofessional teamwork. Multi-centre prospective trials are required to develop a guideline of optimal treatment for the disease entity, particularly the recurrence.

## Highlights

• High-grade endometrial stromal sarcoma (HG-ESS) behaves in a more aggressive manner with a poorer overall prognosis than low-grade ESS.
• Recurrence develops in one-half to two-third of patients with High-grade endometrial stromal sarcoma.
• The major therapeutic procedure of patients with single site recurrent HG-ESS is primarily surgical resection.
• Interdisciplinary and interprofessional teamwork are important in management of recurrent HG-ESS.

## 1 Introduction

Endometrial stromal sarcoma (ESS) is a rare tumor that accounts for 0.2 % of uterine malignancies and 20 % of uterine sarcomas. The current World Health Organization recognizes 4 categories of endometrial stromal tumor: endometrial stromal nodule, low-grade endometrial stromal sarcoma (LG-ESS), high-grade endometrial stromal sarcoma (HG-ESS), and undifferentiated uterine sarcoma [1]. HG-ESS is a rare pathological type of uterine sarcoma and often misdiagnosed as uterine leiomyoma or endometrial carcinoma prior to operation due to a lack of characteristic imaging and clinical manifestations. Information regarding the natural course, prognostic factors, and optimal treatment for HG-ESS is currently limited [2,3].

Over 80 % of affected patients have advanced stage and recurrences occur within a few years of initial presentation. Due to the overall aggressive biologic characteristics of the tumor, most patients undergo cytoreductive surgery (CRS) and multimodality therapy [4]. Here, we present a case of recurrent HG-ESS in the abdominal wall. The patient was treated with wide excision and abdominal reconstruction surgeries. We describe the case presentation according to the SCARE guideline [5].

## 2 Patient information

A 55-year-old, Para 2, woman with BMI of 24.6, was referred to our gynecological clinic at a public national general hospital because of her pathological result after she had undergone total hysterectomy and bilateral salphingoophorectomy at a private hospital. Her pathologic examination revealed high-grade endometrial stromal sarcoma. Prior to the surgery, she had a diagnosis of uterine fibroid with history of post-menopausal bleeding. She had been menopausal for 2 years. Clinical finding and imaging study at that time revealed no site of metastases. She then received adjuvant therapy, which incorporated chemotherapy regimens of Carboplatin (AUC-6) and Paclitaxel (175 mg/m^2^). After six series of chemotherapy, though she was sensitive to chemotherapy, she started to develop bone marrow suppression, nausea and vomiting and hair loss; however, her liver and kidney functions were not affected. The patient was followed-up regularly and she remained in a good condition. Five months after receiving chemotherapy, she complained a rapid abdominal enlargement. She had no history of any systemic disease or malignancy in her family.

## 3 Clinical findings

**Fig. 1:**
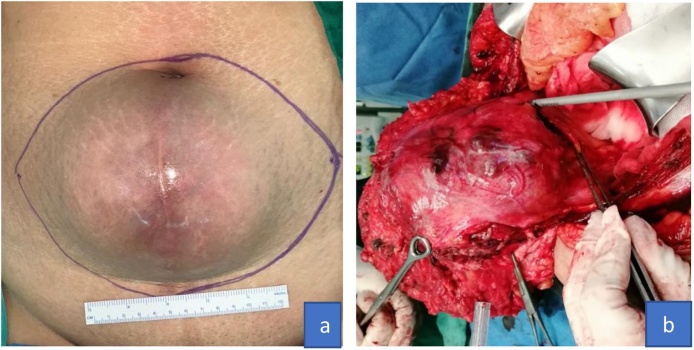
a. Solid mass as high as navel, b. Wide excision of the mass.

While performing abdominal palpation, we found a solid mass at the level of the navel (size: 12 × 15 × 10 cm) with smooth surface and the mass was mobile (Fig. 1). Gynecologic examination revealed a normal vaginal stump. No mass was found in pelvic cavity.

## 4 Timeline

The sequence and order of events in the patient's history and presentation are shown in Table 1.

**Table 1:** Timeline.

Date	Information
June 2018	Post menopausal bleeding, diagnosed uterine leiomyoma. Underwent laparotomy total hysterectomy and bilateral salpingoophorectomy at private hospital. Pathology Anatomy result: high-grade endometrial stromal sarcoma. Referred to our hospital.
September 2018	Whole abdomen MRI: multiple solid mass with necrotic component in left adnexa and middle part of abdominal subcutis, hepatomegaly with cystic metastasis lesion in segmen 2 and 6 of liver. Metastatic nodule in segmen 8 of liver, simple cyst on right kidney (Bosniak Type I). Paraaortic lymph nodes enlargement.
September-December 2018	Adjuvan chemotherapy for 6th series: Carboplatin (AUC-6) and Paclitaxel (175 mg/^m2^).
February 2019	Whole abdomen MRI: visible no mass and pathologic enhancement, nor pelvic and para-aortic lymph nodes enlargement, simple cyst on segment 2 and 6 liver, nodule on segment 8 liver correspond to hemangioma. No metastatic lesion in the liver. Simple cyst on inferior pole of right kidney (Bosniak Type I).
June 2019	Abdominal enlargement with rapid growth.
August 2019	Whole abdomen MRI: multiple solid mass in abdominal wall size 4 × 4 cm. The mass was infiltrated rectus abdominis muscle and also protruded in abdominal cavity.
September 2019	Wide excision of the tumor and abdominal wall reconstruction.

## 5 Diagnostic assessment

Chest X-Ray showed no abnormality in the lung. Ultrasound examination revealed a cystic mass with a thick wall sized 4.82 × 3.43 × 4.81 cm with an infiltration deep into fascia; however, it had not reached peritoneum. Magnetic Resonance Imaging (MRI) confirmed multiple solid masses in the abdominal wall, which were 4 × 4 cm in size. The masses infiltrated rectus abdominis muscle and also protruded into abdominal cavity. We performed that imaging examination 1 month before the wide excision and abdominal wall reconstruction. The patient was diagnosed with recurrent HG-ESS in the abdominal wall.

## 6 Therapeutic intervention

**Fig. 2:**
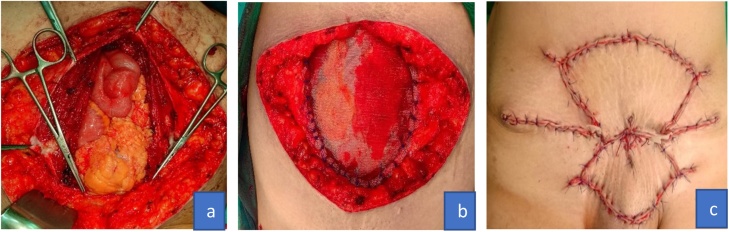
a. Abdominal wall defect, b. Abdominal mesh insertion, c. After reconstruction surgery.

A wide excision and abdominal wall reconstruction surgeries were subsequently performed. The surgical procedures were performed by a gynecology-oncologist. During the surgeries, we found a tumor in the abdominal wall sized 15 × 12 × 5 cm (cranio-caudal x, lateral-lateral x antero-posterior). The tumor had infiltrated the Scarpa’s fascia but did not infiltrate rectus abdominis muscle. When the peritoneal cavity was exposed during the surgery, the tumor protruded into the right lateral peritoneal wall. Enlargement of right parailiac lymph nodes sized 2 cm were palpable. We performed wide excision and frozen section analysis with margins of each section at 12, 3, 6, 9 o’clock positions. The frozen section revealed negative tumor infiltration. Moreover, we also performed debulking of right parailiac lymph nodes, which was then continued with mesh insertion and abdominal wall reconstruction surgery. The abdominal wall reconstruction surgery was performed by a gynecologist-oncologist who collaborated with a plastic and reconstructive surgeon. The plastic and reconstructive surgeon used Keystone flap technique (Fig. 2).

## 7 Follow-up and outcomes

The tumor was brought to the Histopathologic Department. The result conﬁrmed endometrial stromal sarcoma. Margin on cranial, distal, right and left lateral were negative for tumor infiltration. Skin margin on cranial, distal, right and left lateral were negative for tumor. For the right pelvic lymph nodes were negative for tumor.

We gave no adjuvant therapy for this lady. We proceed to do close observation every month for first 3 month. And then every two months for next 6 months. There was no complaint and healing process of the reconstruction wound was good after 6 months follow-up.

## 8 Discussion

ESS is characterized by cells that resemble those of the endometrial stroma during the proliferative phase of the menstrual cycle and have traditionally been classified as either low-grade or high-grade. Tumours with a mitotic count of less than 10 mitoses/10 high power field are considered low-grade, while those with higher mitotic counts are considered high-grade. High-grade ESS (HG-ESS) behaves in a more aggressive manner with a poorer overall prognosis than the low-grade ESS [6,7].

Most patients with ESS are diagnosed with at the early stage of the disease with about 86 % of cases presenting uterine-confined malignancy; for these patients, total hysterectomy and peritoneal cavity exploration remains the therapeutic cornerstone [[8], [9], [10]].

Recurrence develops in one-half to two-third of patients with HG-ESS. It usually includes multiple lung metastases, peritoneal metastases, and/or local recurrences. Recurrence may be attributed to growth stimulus to residual tumor cells by estrogen. After oophorectomy, estrogens produced by peripheral tissues or exogenous administration in the form of hormone replacement therapy may be a reason for recurrence. There is currently no standard therapy for patients with recurrent disease [7]. Due to the rarity of HG-ESS, it is difficult to conduct prospective randomized clinical trials to determine the optimal treatment regimen. Treatment has been deﬁned by the experience gained from retrospective case series and case reports. The major therapeutic procedure of patients with single site recurrent HG-ESS is primarily surgical resection; however, standard treatment including radiotherapy and chemotherapy has not been established [10].

Our case demonstrates that surgical resection can achieve free margin on each dimension of the tumor. Due to rarity of the case and concept of endometrial cellular behavior, also after discussing with patient, we did not give any adjuvant therapy after operation.

## 9 Conclusion

HG-ESS is a rare uterine malignancy, which is rarely diagnosed preoperatively. Many patients will develop disease recurrence. Our case report has described a patient with recurrent HG-ESS in the abdominal wall who had successful treatment. We recommend the treatment procedure; however, multicentre prospective trials are needed to determine optimal therapy for this disease entity in the recurrent setting.

## Declaration of Competing Interest

The authors declare no conflict of interest in preparing this article.

## Sources of funding

The authors did not receive any specific grant from funding agencies in the public, commercial, or not-for-profit sectors.

## Ethical approval

The ethical approval was not required for this case report.

## Consent

Written informed consent was obtained from the patient for publication of this case report and accompanying images. A copy of the written consent is available for review by the Editor-in-Chief of this journal on request.

## Author’s contribution

**Sigit Purbadi**: conceptualization, methodology, validation, formal analysis, investigation, resources, data curation, writing-original draft, writing-review and editing, visualization, supervision, project administration, funding acquisition study concept, data collection, data interpretation, and writing the paper.

**Kade Yudi Saspriyana**: methodology, formal analysis, investigation, data curation, writing-original draft, visualization.

## Registration of research studies

None.

## Guarantor

Sigit Purbadi.

## Patient perspective

Interdisciplinary and interprofessional teamwork are important in the management of recurrent HG-ESS.

## Provenance and peer review

Not commissioned, externally peer reviewed.
